# *In vivo* evaluation of a polyester and fiberglass composite intramedullary nail for femoral osteosynthesis in calves

**DOI:** 10.7717/peerj.16656

**Published:** 2024-02-08

**Authors:** Sérgio Silva Rocha Junior, Mayara G. Corrêa, Lucas A. Dias, Marcos Paulo Antunes de Lima, Suzane L. Beier, Leopoldo Paolucci, Luiz Alberto do Lago, Estevam B. Las Casas, Rafael R. Faleiros

**Affiliations:** 1 Equinova Research Group, Universidade Federal de Minas Gerais, Belo Horizonte, MG, Brazil; 2 Unifenas, Universidade José do Rosário Vellano, Alfenas, MG, Brazil; 3 Escola de Veterinária, Pontifícia Universidade Católica de Minas Gerais, Belo Horizonte, MG, Brazil; 4 Structural Engineering Department, Universidade Federal de Minas Gerais, Belo Horizonte, MG, Brazil

**Keywords:** Fracture, Femur, Composite, Polyester, Fiberglass

## Abstract

The objective of this study was to test a composite of polyester resin and fiberglass in the form of an intramedullary nail for osteosynthesis of femoral fractures in calves. The methodology was established based on a previous study that used a bovine femur finite element model to simulate fractures, which were then stabilized by the same nails as proposed in this study. General anesthesia was induced in six calves followed by fracture creation *via* an oblique incision in the middle third of the femoral diaphysis, and osteosynthesis was immediately performed by retrograde insertion of the composite nail. Locking was achieved by drilling the bone and nail without using a jig and introducing two stainless steel screws proximal and two distal to the fracture line. Five of the six calves achieved complete fracture healing after 60 days. No signs of incompatibility or toxicity of the composite were observed. However, limitations were observed during the surgery, such as difficulty in drilling the nail and trimming the remainder portion of the nail that extended beyond the length of the bone. Small fragments produced by these maneuvers were considered irritating to soft tissues during the postoperative period. It was also found that small cracks in the nail tended to propagate in the form of longitudinal fractures. In conclusion, an intramedullary nail made of polyester resin and fiberglass (a low-cost and easy-to-acquire material) was considered biocompatible and capable of allowing bone healing of femoral fractures in young cattle. However, the development of solutions for the reported limitations is crucial prior to recommending the proposed composite for clinical use.

## Introduction

Long bone fractures in production animals continue to pose a challenge for veterinary medicine. The development of new fracture management techniques and metallic implants in the last few decades has contributed significantly to advances in the treatment of fractures in horses. However, due to the high costs associated with such options, these are not considered feasible for large animals such as dairy or beef cattle. Consequently, the search for increasingly lightweight, resistant, low-cost, and biocompatible materials has intensified over time (Van der Elst, 1999).

Polymers have been progressively tested and incorporated as implants in surgical procedures, with an emphasis on orthopedics and tissue reconstruction. Polymeric materials would be more suitable due to their mechanical properties, which can closely mimic bone, thus avoiding the undesirable effects associated with metallic materials, such as the ‘stress shielding’ phenomenon (Rodrigues et al., 2012). Polypropylene is the most commonly used biocompatible material as a mesh in the repair of abdominal cavity injuries in humans, since it can remain in direct contact with the viscera due to its low reactivity, adding to its advantages of high strength and low cost (Goissis et al., 2001). De Marval (2006) tested polypropylene in the form of a locked intramedullary nail for the treatment of humerus fractures in calves. The results were satisfactory and promising, as this newly developed system allowed bone repair in the evaluated calves without showing any signs of tissue rejection. However, the same study suggested other types of polymers to be tested in this system to improve its efficacy, since polypropylene was not fully effective in maintaining the congruence of bone fragments during the immediate postoperative period. Based on the results of this study, the UFMG Biomechanics Research Group made several efforts to develop solutions applicable to long bone fractures in young cattle. Rodrigues et al. (2012) and Gomidis et al. (2017) developed a finite element model for testing femoral fractures in young cattle, which was validated *in vivo* by Spadeto in 2009. Although these studies did not find an appropriate polymer, since interlocking polyacetal, polyamide, and polypropylene nails failed to allow femur osteosynthesis in calves (Spadeto et al., 2010; Spadeto, Brito & Carvalho, 2011; Rodrigues et al., 2012), the development and validation of the computational model was fundamental for the progress of research in this area as several subsequent studies have utilized this model.

The search for new biomaterials with superior characteristics to those of single polymers led to the evolution of composites by combining different types of materials. For instance, the association of polyester resin with fiberglass offers high mechanical strength, strength-to-weight ratio, chemical resistance, as well as other favorable mechanical properties. A recently published computer simulation study demonstrated that polyester resin associated with fiberglass in the form of a locked intramedullary nail had the potential to withstand mechanical loads in femur fractures in young cattle (Paolucci et al., 2018). The present study aimed to test *in vivo* the effectiveness of a fiberglass polyester interlocking nail in the immobilization and bone healing of experimental femur fractures in calves.

## Materials & Methods

Six male Holstein calves, aged between four and six months and mean weight of 61 ± 15 kg, were used in this study. This study was approved by Committee for Ethics in the Use of Animals (CEUA/UFMG) protocol no. 343/2013. After carrying out a physical exam and hematological and biochemical tests to verify their health status, the calves were kept in 4 m^2^ pens with shavings bedding, where they received commercial feed, hay and water to meet their nutritional needs.

To minimize the use of animals, the ability of the test bar to withstand the mechanical loads characteristic of a calf under static or dynamic conditions was previously evaluated and confirmed through a computer simulation study as described previously (Paolucci et al., 2018).

### Intramedullary nail fabrication

A polyester resin with continuous unilateral long fiberglass reinforcements in the form of a rod was produced by the pultrusion process, which involves heating glass fibers soaked in resin (Rajak et al., 2019).

### Anesthesia and surgery for fracture and nail insertion

After a 36-hour fasting period, premedication was performed with xylazine (0.05 mg/kg, IV), general anesthesia was induced with propofol (4 mg/kg, IV), and the animals were intubated and connected to a circular valve system. Anesthesia was maintained with isoflurane diluted in 100% oxygen, with the minimum alveolar concentration (MAC) adjusted according to the anesthetic requirement. Blood oxygen level and mean arterial pressure were monitored during all procedures, with no need to include additional drugs to this protocol.

Once on the surgery table and after surgical preparation, a skin incision was made centered on the femur diaphysis and tissues were bluntly dissected to expose the femur as described by Milne & Turner (1987). Thereafter, fracture was created in the caudo-proximal to cranio-distal direction with a wire saw at an angle of 30° to the longitudinal axis of the middle third diaphysis.

**Figure 1:**
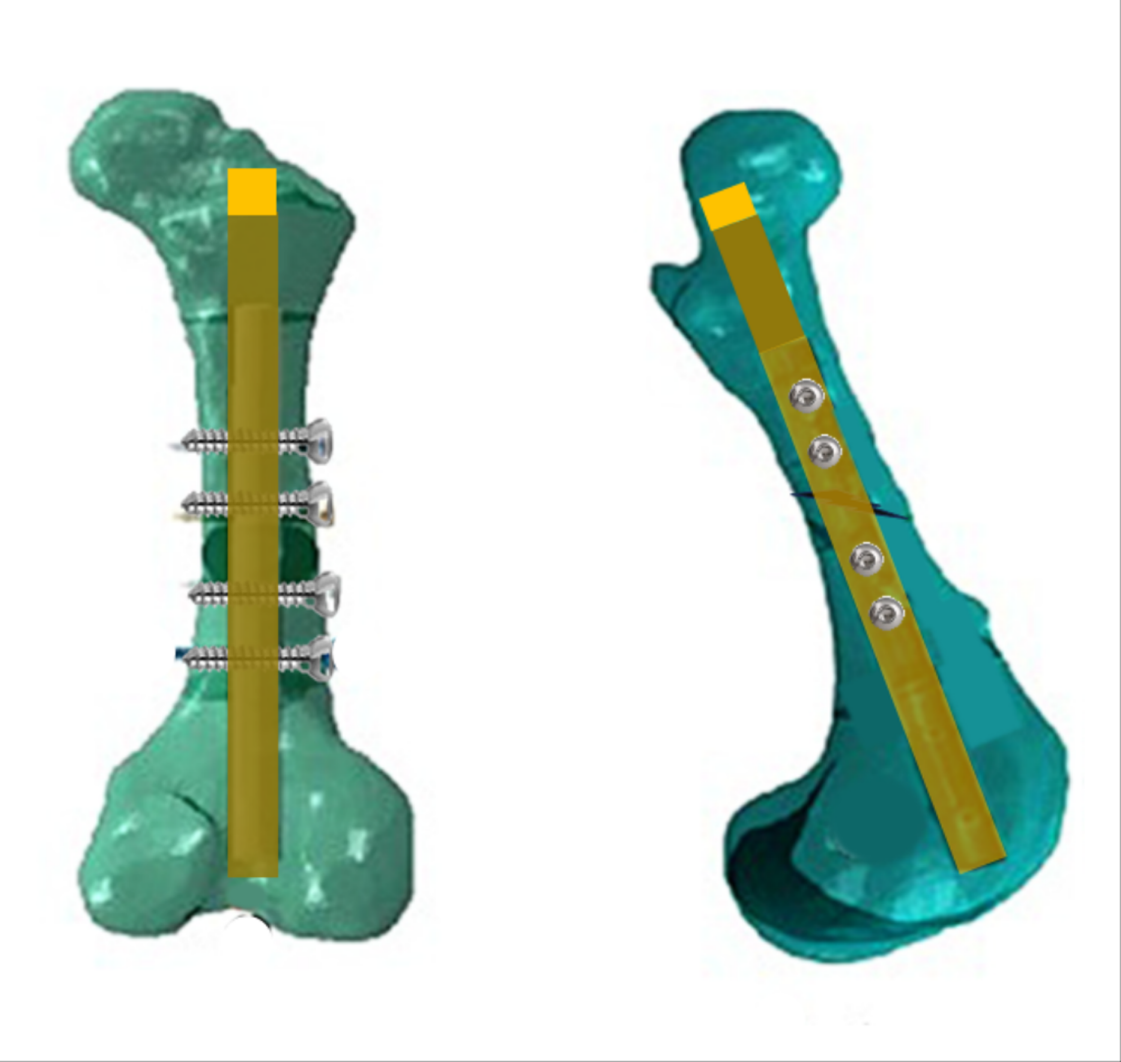
Illustration of femoral osteosynthesis. Schematic illustration of a calf femur with a transverse fracture immobilized with polyester and fiberglass intramedullary nail, locked with two distal and two proximal transverse stainless cortical screws. Caudal view in the left and lateral view in right.

Fracture reduction involved the retrograde application of a previously autoclaved composite nail immediately after the defect was created. This application was tailored to match the thickness of the medullary canal, specifically aiming for a minimum of 80% of the diameter of the medullary canal in the diaphysis. Before inserting the nail, a drill was used to expand the circumference of the medullary canal in both directions. However, in the proximal fragment, additional drilling was carried out to create an opening in the trochanteric fossa of the femur. Subsequently, nail locking was achieved by drilling the bone and nail without the use of a jig or radiographic guidance and introducing two stainless steel screws proximal and two distal to the fracture line. At the end of the procedure, the remainder portion of the nail that extended beyond the length of the bone at the trochanteric fossa was trimmed using a saw wire. The outcome of the femoral osteosynthesis using the described interlocking nail procedure is illustrated in Fig. 1.

Following surgery, antimicrobial therapy with procaine benzylpenicillin, potassium benzylpenicillin and streptomycin (20,000 IU/kg), and gentamicin (6.6 mg/kg) was administered every 24 h for five days. For pain control, tramadol (3 mg/kg, IM, BID) was administered for four days, and meloxicam (0.6 mg/kg, IV, SID) was given on the first postoperative day, followed by half of this dose (0.3 mg/kg) for the next six days.

### Postoperative evaluation

Radiological examinations were performed before surgery, in the immediate postoperative period, and 15, 30, 45, and 60 days after surgery. Craniocaudal and lateral oblique views at power settings of 90 kV and 2.5 mAs were used to verify the position of the implants, the formation of the bone callus and possible complications, such as site instability, implant migration and osteolytic and periosteal changes. A portable X-ray emitter was used (#1060 HF, ManoMedical, Taden, France), and the obtained images were digitized using a computerized radiology system and stored in a recording unit (CR Regius 110, Konica Minolta, Los Angeles, CA, USA).

Additionally, ultrasound examinations were performed at 15, 30, 45, and 60 days postoperatively using a device with linear as well as convex probes to assess the formation of bone callus, the overlap between the bone fragments, and the formation of seroma. During examination, the animals were restrained in a quadrupedal position. To assess the fracture site, the probe was positioned in the middle third of the femur and then the nail insertion point was evaluated. A scoring system ranging from zero to three was created for these evaluations (Table 1).

**Table 1:** Ultrasound evaluation score. Scores for ultrasound evaluation of the incision and nail wounds in calves subjected to femoral osteosynthesis.

**Score**	**Ultrasound changes**
0	No changes
1	Slight irregularity around the focus
2	Discrete fluid accumulation
3	Severe fluid accumulation

During the 60-day follow-up period, blood samples were collected weekly from the jugular vein and conditioned in tubes containing EDTA for performing complete blood cell counts and determining the concentrations of plasma total proteins and fibrinogen (Kaneko, Harvey & Bruss, 1997). Every 15 days, blood samples were collected in a tube containing a clot activator and used to determine the concentrations of alanine aminotransferase (TGP), aspartate aminotransferase (TGO), creatinine, gamma glutamyl transferase (GGT), glucose, globulin, albumin, urea, as well as the albumin to globulin ratio and albumin to alkaline phosphatase (AF) ratio. UV enzymatic methodology was used to obtain the values of TGP, TGO, GGT, and AF. Total proteins and albumins were analyzed *via* infrared (IR) calorimetry, whereas kinetic method was used for creatinine measurement. Furthermore, glucose values were obtained through the enzymatic IR method and mathematical difference between the total proteins and albumins was used to obtain the values of globulins.

### Bone biopsy

All calves underwent bone callus biopsy after 30 and 60 days of surgery. The samples were obtained using a bone marrow aspiration needle (Jamshidi, 8G) inserted in the middle third of the diaphysis on the lateral aspect of the fractured femur. After sedation with 2% xylazine (0.05 mg/kg) and local anesthesia with 2% lidocaine, the biopsy site was surgically prepared using chlorhexidine followed by 70 alcohol solution topically. After confirming the position of the needle on the bone callus by ultrasound and radiography assessment, the needle was introduced through the callus of the lateral cortex of the femur to secure the sample.

Thereafter, the bone fragments were fixed in 10% buffered formalin and subsequently decalcified in a solution containing 250 ml formic acid (90%), 100 g sodium citrate, and 750 ml distilled water. After complete decalcification, these fragments were processed using routine laboratory techniques, embedded in paraffin wax, cut in a microtome at section thickness of 5 µm, stained with hematoxylin and eosin, and examined under a light microscope. A scoring system was used to assess bone callus formation in all cases (Table 2).

**Table 2:** Histological socre. Scores for histological interactions in the fracture focus of calves subjected to femoral osteosynthesis with a composite intramedullary.

**Score**	**Histological findings in the fracture focus**
1	Fibrous tissue (fibrous callus)
2	Mainly fibrous tissue and small amount of cartilaginous tissue
3	Similar amounts of fibrous tissue and cartilaginous tissue
4	Only cartilaginous tissue
5	Mainly cartilaginous tissue and small amount of immature/primary bone
6	Similar amounts of cartilaginous tissue and immature/primary bone
7	Significant amount of immature/primary bone and small amount of cartilaginous tissue
8	Only immature/primary bone
9	Immature/primary bone and small amount of mature/lamellar/secondary bone
10	Only mature/lamellar/secondary bone

### Data analysis

The time effects on hematological and biochemical variables were analyzed by performing ANOVA in a randomized block design, and means were compared using the Tukey’s test. The Kruskal Wallis, Dunn and Wilcoxson tests were used to compare the ultrasonographic and biopsy grades over time. Results were considered significant at *P* < 0.05.

## Results

### Surgical technique for fracture induction and osteosynthesis

Approaching the left femur through the lateral aspect of the thigh allowed easy access to the middle third of the femur, enabling fracture creation and retrograde application of the intramedullary nail. However, the application of locking screws at the ends of the diaphysis, as recommended by the computer simulation study (Paolucci et al., 2018) was not possible due to the limited surgical exposure promoted by the presence of the origin and insertion of the biceps femoris, gastrocnemius, and vastus lateralis. Nevertheless, the nail locking procedure was effective in achieving proper alignment of the bone fragments and ensuring immediate and effective immobilization of the fracture site in all calves.

### Postoperative evaluation

Two days after surgery was performed on the first calf, instability was observed at the fracture site and radiographic evaluation revealed a fracture of the intramedullary nail. The animal was reoperated, and it was found that perforations in the distal portion of the nail were not centralized. This case was considered a technical failure, so a new nail was correctly inserted and the calf was retained in the experiment for a second trial.

Site instability and nail fracture were also observed in the sixth calf, 21 days after surgery. In this instance, the failure was attributed to the nail material, despite correctly executed perforations. To address this bone fracture, a dynamic compression plate and metallic screws were used. This case was considered as a mechanical failure of the composite due to a longitudinal fracture in its center, seemingly initiated by a small crack during the drilling process.

Radiographic follow-up during the postoperative period allowed us to observe the evolution of bone consolidation during the experimental period. At 15 days after surgery, it was possible to verify the coaptation between the bone fragments, but there were no radiographic signs of the developing callus, which began to appear after 30 days of surgery. At the 45th day, the bone callus was clearly noticeable; however, the fracture line was still visible. At the 60th day after surgery (end of the experimental period), the callus was well developed, and the fracture line was not visible, characterizing bone consolidation.

A few complications were encountered during the follow-up. In the third calf, misalignment of the bone fragments and migration in two of the locking screws was observed 15 days after the operation, but without complete loss of congruence. However, there was no instability at the fracture site at any time, and the fracture consolidated after 60 days. In addition, after 30 days of surgery, a longitudinal fracture of the distal part of the nail was observed in the fifth calf upon radiographic examination. However, this change did not affect the evolution of consolidation. Except for calf six, which was removed from the experiment, all calves presented fracture healing at the end of the experiment.

**Figure 2:**
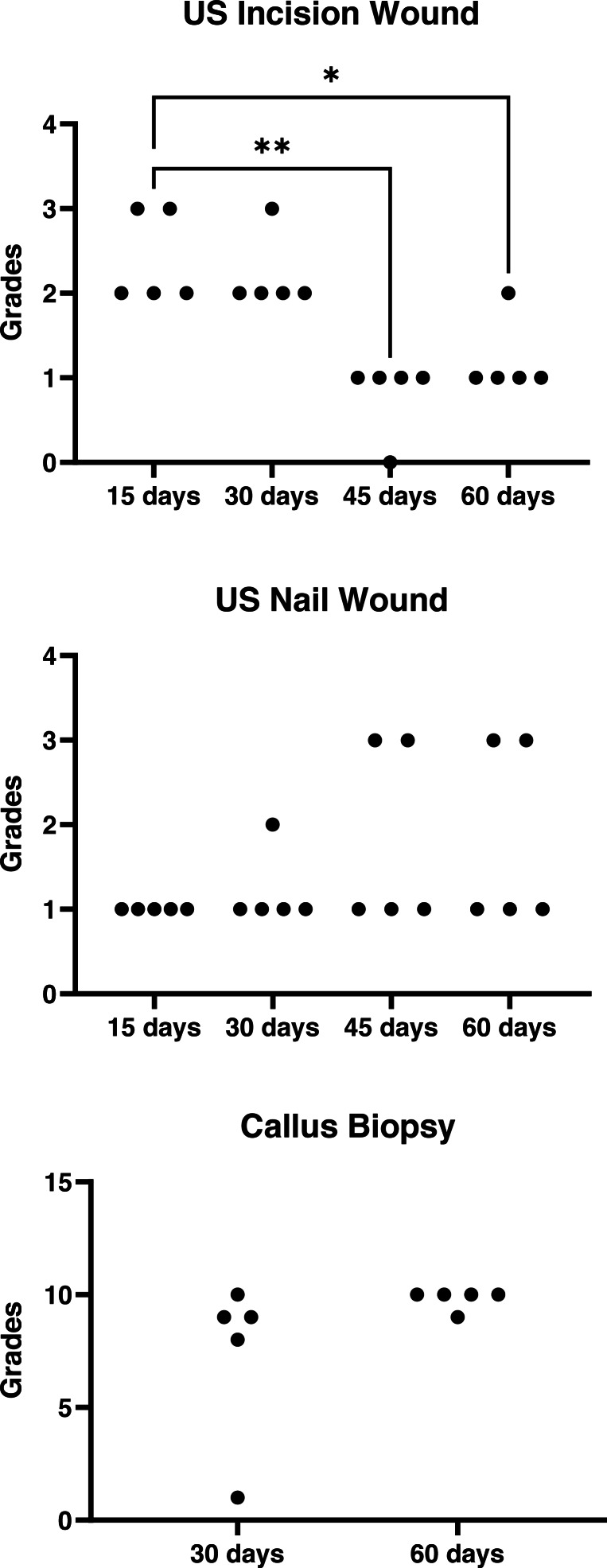
Ultrasonographic and histological grades graphs. Wound ultrasonographic (US) and biopsy histological grades in the postoperative period of calves subjected to a femoral fracture immobilized with a composite intramedullary nail.

Ultrasound examination data were presented in Fig. 2. There was a decrease in lesion grades over the time in the incision wound site (*P* = 0.002) that did not happened in the nail wound site (*P* = 0.36). At the 15th day hyperechoic areas were seen between the bone fragments, compatible with the beginning of fibrous callus formation. In addition, circular anechoic areas characteristic of seroma formation were observed in all animals. At the exit point of the nail between the greater and lesser trochanters, irregular hyperechoic areas were observed around the nail and in one case an anechoic area indicative of seroma was also visualized. At the same time, measurements of the distance between the fractured bone fragments were performed, which ranged from 0.55 mm to 0.18 mm. On the 30th postoperative day, the findings were similar to those found during the previous evaluation; however, it was no longer possible to measure the distance between the bone fragments due to the formation of bone callus in all cases. On the 45th day, seromas were no longer visible at the fracture site, but they were still present at the nail exit point in two animals. The primary observation made during the last ultrasound evaluation of the fracture site was a hyperechoic area covering the two bone ends of the femoral shaft, characterizing a well-defined bone callus. The seromas that were evident at the nail exit points at day 45 in the two cases described above were still present.

A few other complications were also observed during the experimental period. Calf number five presented hyperextension of the metatarsal phalangeal joint of the right hind limb, 14 days after the surgical procedure. In addition, calf number one had a floating area over the exit point of the nail, which was revealed on ultrasound as fluid accumulation in the subcutaneous tissue surrounding the proximal end of the nail. Subsequently, aspiration puncture was performed to obtain a reddish liquid with high viscosity, which was sent for culture and cytology. Although cytological analysis of the aspirated fluid showed presence of degenerated neutrophils, no bacterial growth was detected during culture evaluation. The fluid was drained *via* a skin incision, followed by daily washings with lactated Ringer’s solution and aqueous chlorhexidine (0.2%), until wound healing.

### Hematological and biochemical tests.

Blood work results (Table 3) indicated leukocytosis associated with neutrophilia was observed exclusively in the first postoperative week (*P* < 0.05). In the third week, there was a transient decrease in packed cell volume. Additionally, an increase in plasma fibrinogen levels was observed from the first postoperative week, which returned to normal by day 60. Slight thrombocytopenia was noted during the first week, which was compensated by thrombocytosis in the fourth and fifth weeks. The eosinophil counts remained within normal levels throughout the duration of follow-up; however, there was a slight increase, especially in the sixth, seventh, and eighth postoperative weeks.

**Table 3:** Hematological tests data. Means and standard deviation of hematological tests from calves subjected to femur fracture and osteosynthesis with composite intramedullary nail.

**Variable**			**Week after surgery**							
		**Baseline**	**1**	**2**	**3**	**4**	**5**	**6**	**7**	**8**
**Erythrocytes**	Mean	7.25	7.02	6.53	6.63	6.04	6.33	5.44	6.29	6.83
(10^9^/uL)	SD	1.98	1.00	0.93	1.71	1.87	2.64	1.79	1.44	1.41
**Hematocrit**	Mean	25.66	26.33	28.50	25.16	27.33	26.16	26.50	27.16	28.83
(%)	SD	4.84	5.31	5.54	7.19	4.45	5.63	4.27	2.78	2.40
**Hemoglobin**	Mean	8.80	9.34	9.52	9.78	9.35	8.73	8.61	9.43	9.48
(d/dL)	SD	1.83	1.58	1.74	2.13	1.77	2.27	2.15	1.55	0.97
**Leukocyte**	Mean	13,305	11,608	9,533	9,066	9,450	10,438	10,065	9,923	10,358
(/uL)	SD	6,567	4,901	3,112	3,587	3,984	4,418	3,727	3,400	2,421
**Neutrophils**	Mean	3,405	5,773	2,831	2,988	3,277	3,093	3,638	3,774	4,226
(/uL)	SD	1,809	3,578	1,534	1,454	2,074	1,548	1,706	1,523	1,190
**Lymphocyte**	Mean	5,845	4,933	5,993	4,191	5,830	6,824	5,551	5,272	5,061
(/uL)	SD	2,831	2,792	2,367	2,064	2,003	3,572	2,282	1,700	1,142
**Eosinophils**	Mean	57^a^	39^ab^	145^ab^	72^ab^	88^ab^	77^b^	177^ab^	269^b^	321^b^
(/uL)	SD	54	62	109	97	127	85	116	180	153
**Monocytes**	Mean	236	862	654	313	348	476	754	606	750
(/uL)	SD	230	659	655	274	196	72	296	298	134
**Platelets**	Mean	453,966^a^	173,600^b^	149,233^b^	102,666^b^	78,183^b^	94,250^b^	106,533^b^	102,383^b^	109,550^b^
(/uL)	SD	20,4050	76,526	56,905	19,024	37,788	26,777	13,437	6,872	25,132
**Fibrinogen**	Mean	583^a^	800^ab^	1,033^b^	933^ab^	733^ab^	767^ab^	600^ab^	600^ab^	467^a^
(mg/dL)	SD	306	219	409	163	163	150	179	126	163
**Proteins**	Mean	7.73	7.10	7.76	7.06	7.0	6.80	7.53	7.33	7.4
(g/dL)	SD	0.95	1.20	1.05	0.85	0.81	0.73	0.41	0.20	0.33

**Notes.**

Means followed by the same letter do not differ (*P* < 0.05).

About the biochemical tests (Table 4), an increase in the TGP values was observed from day 0; however, the levels remained within the reference values. The TGO values also remained within the reference values throughout the period of observation, with a decrease around day 60. With respect to total proteins, hyperproteinemia due to increased albumin levels was observed at all times (Table 3).

**Table 4:** Biochemical tests data. Means and standard deviation (SD) of biochemical tests from calves subjected to femur fracture and osteosynthesis with composite intramedullary nail.

**Variable**			**Days after surgery**			
		**Baseline**	**15**	**30**	**45**	**60**
**TGP**	Mean	14.55^a^	14.91^a^	18.77^b^	20.06^b^	19.41^b^
(U/L)	SD	3.52	2.81	3.51	3.44	3.83
**TGO**	Mean	50.78^ab^	51.38^ab^	53.44^a^	45.45^ab^	43.50^b^
(U/L)	SD	5.64	5.35	11.68	6.15	6.99
**GGT**	Mean	19.99	18.29	21.38	21.71	20.43
(U/L)	SD	14.20	7.89	6.06	2.45	2.57
**Alkaline phosphatase**	Mean	90.94	93.76	106.28	112.08	125.01
(U/L)	SD	22.94	20.12	27.31	71.04	47.13
**Glucose**	Mean	67.62	73.35	64.0	75.55	75.73
(mg/dL)	SD	21.73	14.97	14.28	10.81	14.0
**Total proteins (g/dl)**	Mean	7.62^a^	7.63^a^	8.82^ab^	9.68^b^	8.91^ab^
(g/dL)	SD	1.78	1.27	0.87	0.95	1.18
**Globulins**	Mean	4.58^a^	4.51^a^	5.22^ab^	6.39^b^	5.59^ab^
(g/dL)	SD	1.68	1.27	1.09	1.05	1.31
**Albumin**	Mean	2.93^a^	3.12^ab^	3.44^b^	3.43^ab^	3.33^ab^
(g/dL)	SD	2.027	0.15	0.51	0.18	0.18
**Globulin albumin**	Mean	0.61	0.74	0.57	0.55	0.62
(ratio)	SD	0.32	0.24	0.14	0.11	0.17
**Creatinine**	Mean	0.61	0.66	0.72	0.75	0.73
(mg/dL)	SD	0.24	0.21	0.15	0.11	0.37
**Urea (mg/dl)**	Mean	30.52	30.32	30.77	33.51	33.23
(mg/dL)	SD	7.07	6.07	9.33	15.09	10.75

**Notes.**

Means followed by the same letter do not differ (*P* < 0.05).

### Bone biopsy

**Figure 3:**
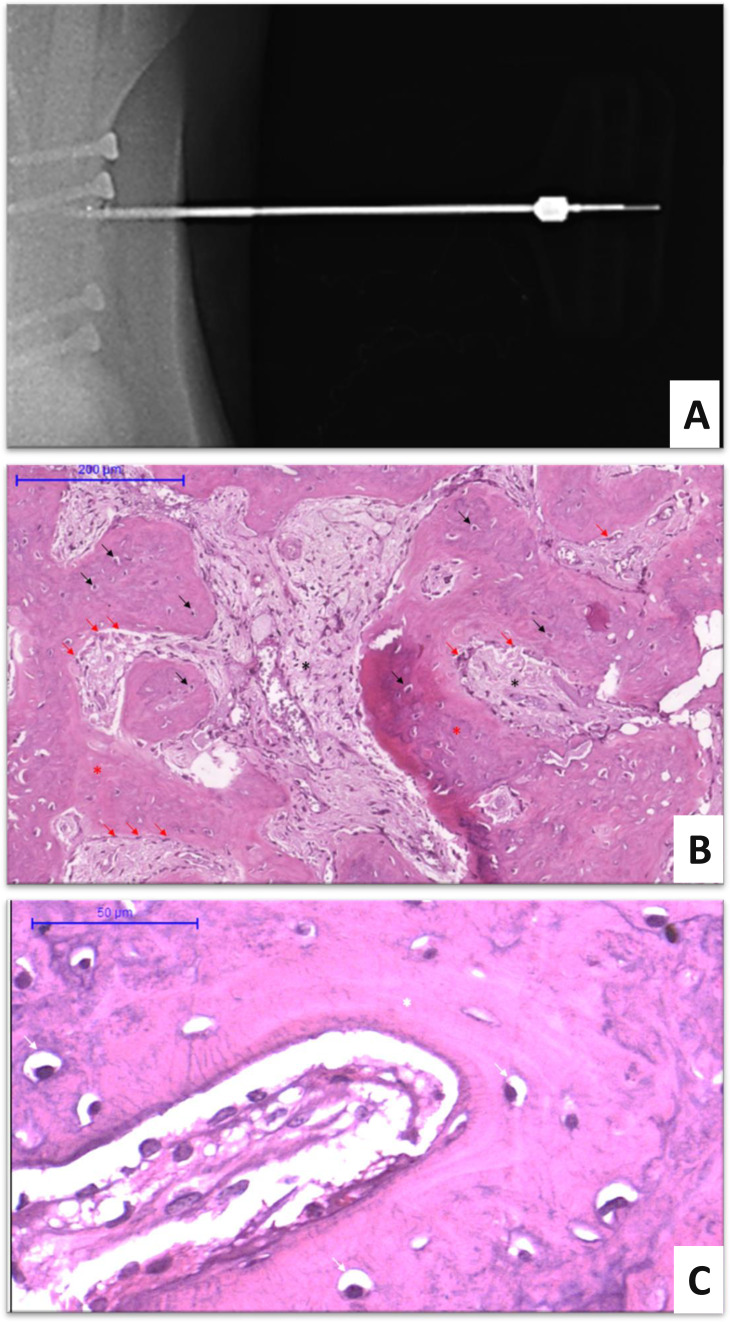
Biopsy procedure and histological evaluation. Bone biopsy in calves subjected to experimental osteosynthesis of femoral fracture with intramedullary nail of polyester resin and fiberglass. (A) Radiographic image demonstrating the introduction of the Jamishidi needle in the callus. (B) Photomicrograph of bone callus biopsy 30 days after surgery (H&E, 10x). Black arrows indicate the osteocytes, surrounded by the matrix of the primary bone callus (red asterisk). Red arrows indicate osteoblast rhymes. Black asterisks indicate the granulation tissue. (C) Photomicrograph of bone callus biopsy 60 days after surgery (H&E, 10x). White arrows indicate the osteocytes surrounded by the secondary bone matrix (lamellar bone).

Although, no statistical differences (*P* = 0.13) were detected in the histological grades obtained at 30 and 60 days (Fig. 2), the results of the histological evaluations were consistent with those of the normal phases of the consolidation process at all time points (Fig. 3). The use of radiography as a confirmation method for positioning of the needle on the bone callus allowed better delimitation of the bone callus and visualization of the screws (Fig. 3A). The use of the Jamshidi needle allowed easy penetration into the bone callus, allowing the acquisition of bone fragments approximately three mm in length. After biopsy collecting, no complications were observed, such as infection at the puncture site or interference in the bone healing process.

## Discussion

In our study, we observed that fiberglass and polyester composite nails demonstrated a capacity to withstand biomechanical forces, facilitating bone callus formation and femoral fracture healing in 5 out of 6 calves. Nonetheless, we encountered challenges both during and after the surgical procedures, which are discussed in the following paragraphs.

The technique of lateral approach to the femur described by Milne & Turner (1987) and used by Spadeto (2009) proved to be easy to perform, allowing good access to the femoral diaphysis for fracture induction and retrograde application of the intramedullary nail. However, the use of this technique did not allow extension of the incision in the proximal and distal directions of the femur because of the origin and insertion of the biceps femoris, gastrocnemius, and vastus lateralis muscles. Thus, there was a limitation in the distance of the screws in relation to the fracture site.

In a previous study of finite elements using three types of locking conditions in the nails (Paolucci et al., 2018), placing the locking screws at both diaphysis extremities, further away from the fracture site proved to provide a better load distribution at the interface between the bone and implant, reducing the risk of failure. Although this limitation was not considered totally decisive, since there was fracture consolidation in five of the six calves, its occurrence was relevant as the failure in the nails always occurred at the interface with the screws.

In a prior study conducted on calves, utilizing the same fracture model as the current study and employing polyacetal nails, there were six cases of fractures occurring in the immediate postoperative period, all at the interface between the nail and screws (Spadeto, 2009). The weakening of polymeric nails due to screw insertion has also been documented in an ex vivo study that used a model of femoral diaphyseal fracture, subjected to flexion tests in a universal testing machine (Spadeto et al., 2008).

It is believed that the screw placement should be performed at 1–3 cm from the fracture site due to the risk of fracture propagation (Seyhan et al., 2013). In the present study, screws were inserted approximately 1 cm away from the fracture site, and no cases of bone weakening were observed during the execution of this technique.

The process of nail drilling during surgery without the use of a jig can be challenging because there is a possibility that the drilling may not align perfectly with the center of the nail, even when the drill is placed precisely in the axial plane of the bone diaphysis. One reason for that is because the medullary canal becomes wider from the center of the diaphysis towards the bone ends. This observation was also made in a study using intramedullary polymeric nails in the humerus of young cattle (De Marval, 2006). A similar disparity in nail diameter concerning the medullary canal at the ends of bones has been documented in humans as well, particularly in the distal part of the femur, where the medullary canal is wider, hindering complete filling with the nail (Ricci, Gallagher & Haidukewych, 2009).

To overcome this problem in the future, the development of a drilling jig to perform perforations and fixation of the locking screws could be of great value, as has been already suggested by other researchers in the past (De Marval, 2006; Spadeto, 2009). Fluoroscopy can also be helpful for screw placement in such cases, but it has the disadvantage of the need of extra equipment and exposing the surgical team and patient to the risks of radiation (Seyhan et al., 2013).

Another limitation of retrograde insertion of the nail into the medullary canal was the impossibility of correctly trimming the remainder of the nail, close to the exit hole in the trochanteric fossa. The presence of the nail outside the medullary canal favored the development of seroma in three of the operated animals and lasted until 60 days postoperatively in two animals, which did not happen in the fracture wound (Fig. 2). However, no difficulties were encountered with respect to healing of the skin, and the skin sutures were removed in all calves at day 15, without any signs of infection or delayed healing.

### Postoperative evaluation

In the present study, one of the calves presented with a misalignment of the bone fragments during radiographic examination. Similar observation was made by De Marval (2006) using the same fracture model in the humerus of calves. Undoubtedly, the oblique angle of the osteotomy line (approximately 30° to the longitudinal axis) contributed to the lack of alignment by favoring sliding between the bone fragments.

In addition to the above stated complication, detachment of cortical screws located in the distal portion of the bone diaphysis was identified in the radiographic images of one of the operated animals. One possible explanation for this observation could be the low bone density of the femoral cortex in young calves (Trostle & Markel, 1996; De Marval, 2006; Rodrigues et al., 2012), leading to poor anchorage of the cortical screws in this case.

The middle-third nail failure that occurred 21 days after surgery was not associated with inadequate screw placement. Factors that could have caused this fragility included an error during nail construction or probably the unidirectional arrangement of the glass fibers in the nail, which would allow any small crack to propagate along its entire length, causing a structural collapse of the rod. This fact must be considered when constructing new composite prototypes, which can be improved using another reinforcement fiber configurations, like bidirectional long fibers or the short-fiber aligned or randomly oriented designs (Rajak et al., 2019).

With the exclusion of one calf with a broken nail, the fractures resolved completely in the other five calves. The scale for union of tibial fractures (Kooistra et al., 2010) was used to confirm bone healing by evaluating the bone callus and fracture line on radiographic images. In this system, bone healing is graded on a scale of 1–3, as follows: (1) the bone callus is absent and the fracture line is visible, (2) the bone callus is present and the fracture line is visible, and (3) the bone callus is present, and the fracture line is absent. This is a significant result, as previous attempts at achieving osteosynthesis using the same model with intramedullary nails of polyacetal and polyamide were considered insufficient in promoting bone healing (Spadeto et al., 2010).

Ultrasound assessment is regarded as a valuable resource for monitoring the bone healing process as it allows the identification of complications, such as seroma formation in the postoperative period. Ultrasound examination allowed us to easily visualize the fracture site, measure the distance between fracture fragments, and evaluate the changes related to nail exit point in the intertrochanteric fossa, as described previously (Spadeto, 2009).

The most noteworthy ultrasound finding of the present study was the identification of fibrous callus formation on day 15, which coincided with the bone repair phase of replacement of the hematoma formed at the time of fracture by fibrovascular tissue rich in collagen fibers (McKibbin, 1978). This demonstrates the advantage of ultrasonography in relation to the use of radiology to monitor the initial process of bone healing in operated calves. The findings of the current study corroborate those of prior studies (Maffulli & Thornton, 1995; Moed et al., 1998), where radiographic assessments did not detect the non-ossified callus formed at the beginning of bone healing, unlike ultrasound, which allowed effective visualization of such changes. Among the disadvantages of using radiography for postoperative follow-up, exposure to ionizing radiation (Wawrzyk et al., 2015) and the impossibility of identifying the initial phase of calcification have also been reported in the past (Craig, Jacobson & Moed, 1999; Wawrzyk et al., 2015).

Analogous to a few previous reports of femoral fracture in calves (Spadeto et al., 2010), calf number five of our study developed metatarsophalangeal joint hyperextension in the collateral limb. According to Câmara et al. (2014) and Mulon (2013), this change is associated with immobilization of the affected limb and uneven distribution of weight on the contralateral limb, causing hyperextension of the metacarpal or metatarsal phalangeal joint due to loosening of the superficial and deep digital flexor tendons. This complication is commonly seen in cases where the bone healing process is delayed or the stall in which the calves are kept in the postoperative period are uncomfortable. In the present case, it was believed to arise due to the weight and behavior of the calf, as the animal had a high body score compared to other calves and remained standing for most of the day, which contributed to the overload on the contralateral limb.

The seroma observed at the free end of the nail in one calf was not associated with any infectious process or foreign body reaction, since the culture report returned negative, and the wound created for fluid drainage healed quickly and completely. It is believed that this occurrence was related to the second osteosynthesis performed in this case. During this procedure, several fragments of the original nail were found between the muscular and subcutaneous tissues. Although a previous biocompatibility study in a rat model revealed a low potential for irritation by the composite (Rocha, 2019), it was discovered that small fragments of fiberglass released during nail drilling or due to fracture can be irritating to the surgeon’s hands during manipulation of the nail.

### Biochemical and hematological tests

An inflammatory response is considered typical of any surgical procedure. The slight leukocytosis observed in the present study was consistent with an inflammatory response caused by the surgical procedure. Furthermore, an increase in the levels of segmented neutrophils in cattle is an indicator of an acute inflammatory response, and these changes remain for a variable duration, depending on the type of tissue injury (Kramer, 2000). Elevation in fibrinogen levels was also consistent with the normal healing process at the surgical site, and this finding was similar to that of Spadeto (2009).

Hyperproteinemia was understood to be associated with elevated globulin levels, since albumin values remained within the reference values. One of the main explanations for hyperglobulinemia is the antigenic stimulus caused by infectious agents (Sulaiman et al., 2010; Esmaeilnejad, Tavassoli & Asri-Rezaei, 2012). Microorganisms of the *Anaplasma* species, an intra-erythrocyte rickettsia commonly transmitted by ticks and hematophagous insects (Farias, 2001), were detected in the blood smears of all calves after surgery. Therefore, anaplasmosis might explain not only the changes in blood globulin levels but also the hematocrit drop during the postoperative period, as it is accepted as one of the primary causes of anemia in bovine calves (Farias, 2001).

Normal levels of eosinophil count and other biochemical parameters during the experimental period provided evidence that the composite nail did not evoke any systemic negative effects. Biocompatibility is the ability of a biomaterial to trigger an appropriate biological response, without causing adverse reactions, such as chronic inflammation, foreign body reaction, or toxicity (Freire et al., 2012).

### Bone biopsy

The bone repair process of fracture resolution follows the initial inflammatory response with the organization of the non-mineralized callus. This phase is characterized by the gradual replacement of the hematoma formed at the time of fracture, which acts as a framework for the genesis of the bone callus. Initially, this hematoma is replaced by fibrovascular tissue containing collagen fibers, which later becomes mineralized to form a temporary or primary bone callus (McKibbin, 1978; Lopez & Markel, 2012). The histological evaluations of the bone biopsies on day 30 of our experiment were consistent with the phase of mineralization and resorption of the cartilaginous callus, that is, the phase of provisional bone callus formation, where the predominant histological finding is the primary bone (Fig. 3B). However, at this time point, one calf presented a predominance of fibrous tissue, indicating a delay in the bone repair process. This animal required a second surgery to replace the nail and subsequent drainage of the proximal region, which explained the histological findings.

Although three of five calves biopsied at day 30 showed signs of osteonecrosis, probably related to the medullar drilling process, the predominant histological finding at day 60 was the presence of lamellar bone (Fig. 3C), which was consistent with the bone remodeling phase of fracture repair. The remodeling phase is the third and largest phase of the bone healing process, during which cartilage tissue is exchanged for bone tissue, which then undergoes remodeling into lamellar bone in addition to resorption of the nonessential callus (McKibbin, 1978; Lopez & Markel, 2012). Thus, the natural process of bone consolidation was restored at day 60. In addition, appearance of primary bone was verified in calf one, demonstrating that there was a recovery from the delay in the bone repair process verified at day 30. In addition to the findings described at day 30 and day 60, no other histological changes were observed that could have suggested a tissue rejection response to the fiberglass and polyester resin composite.

Taken together with the biochemical and hematological results and results of our previous studies using rats (Rocha, 2019), the current findings indicate that the polyester and fiberglass composite has biocompatible properties and can be well tolerated as a bone intramedullary implant in cattle. However, it is worth noting that this composite can be more irritating when fragmented and thus additional research is warranted to evaluate its biocompatibility in the medium and long term.

## Conclusions

The fiberglass and polyester composite nails could resist the biomechanical forces, allowing bone callus formation and femoral fracture healing in young cattle. However, improvements are still need in the prototype like changing the design of the fiber reinforcement to avoid longitudinal fractures and the development of a specific jig that can resolve the problems encountered during the drilling and trimming processes.

The composite was considered biocompatible in this short-term assay. However, further research is needed in this area because evidence suggests that dust and small fragments associated with the composite nail can irritate the tissues.

## Supplemental Information

10.7717/peerj.16656/supp-1 Supplemental Information 1 Raw data

10.7717/peerj.16656/supp-2 Supplemental Information 2 Author Checklist

## Additional Information and Declarations

### Data Availability

The following information was supplied regarding data availability: The raw data are available in the Supplemental File and Fig. 1.
